# The interaction between the major vault protein rs4788186 polymorphism, alcohol dependence, and depression among male Chinese problem drinkers

**DOI:** 10.3389/fpsyt.2023.1111712

**Published:** 2023-07-21

**Authors:** Yuyu Wu, Ke Zhao, Yingjie Chen, Liujun Wu, Feng Qiu, Yuying Yuan, Guanghui Shen, Kexin Wang, Yimin Kang, Yongsheng Jiang, Wei Wang, Li Chen, Yanlong Liu, Xuebo Pan, Fan Wang, Longteng Xie

**Affiliations:** ^1^Zhejiang Provincial Clinical Research Center for Mental Disorders, The Affiliated Wenzhou Kangning Hospital, Wenzhou, China; ^2^School of Mental Health, Wenzhou Medical University, Wenzhou, China; ^3^Lishui Second Affiliated Hospital of Wenzhou Medical University, Lishui, China; ^4^Cixi Biomedical Research Institute, Wenzhou Medical University, Ningbo, China; ^5^Applied Psychology (Ningbo) Research Center, Wenzhou Medical University, Ningbo, China; ^6^School of Pharmaceutical Sciences, Wenzhou Medical University, Wenzhou, China; ^7^Key Laboratory of Psychosomatic Medicine, Inner Mongolia Medical University, Huhhot, China; ^8^The Affiliated Xiangshan Hospital of Wenzhou Medical University, Ningbo, China; ^9^Beijing Hui-Long-Guan Hospital, Peking University, Beijing, China

**Keywords:** alcohol dependence, depression, major vault protein, single nucleotide polymorphism, G × E

## Abstract

**Objective:**

Alcohol use disorder (AUD) is the second most prevalent mental disorder and might be related to depression. Major vault protein (MVP) is a cytoplasmic protein related to vesicle transport. The present study aimed to investigate the interaction between a genetic variant (MVP rs4788186) and depression in adult male Han Chinese with AUD during withdrawal.

**Methods:**

All participants (*N* = 435) were diagnosed with AUD. Alcohol dependence level was measured using the Michigan Alcoholism Screening Test, and depression was measured using the self-rating depression scale. Genomic DNA was extracted from peripheral blood and genotyped.

**Results:**

Hierarchical regression analysis identified an interaction between MVP rs4788186 and alcohol dependence level for depression (*β* = −0.17, *p* < 0.05). Then, a region of significance test was performed to interpret the interaction effect. Re-parameterized regression models revealed that the interaction between MVP rs4788186 and alcohol problem severity fit the strong differential susceptibility model (*R*^2^ = 0.08, *p* < 0.001), suggesting that the AA homozygotes would be more likely subjects with the G allele to experience major depression symptoms.

**Conclusion:**

Carriers of the AA homozygote of MVP rs4788186 may be more susceptible to severe alcohol problems and higher levels of depression during withdrawal.

## 1. Introduction

Alcohol consumption is a significant public health concern, leading to disabilities, poor health, and three million deaths every year. Excessive alcohol use accounts for 5.1% of the global disease burden (1). China has experienced the fastest-rising alcohol consumption in the world over the past few decades, which has been accompanied by significant increases in alcohol dependence and use disorders (2). Alcohol use disorder (AUD) is the second most prevalent mental disorder globally, following depression. It is characterized by a poorly adapted pattern of alcohol use, including repeated and heavy drinking, cognitive impairment, and alcohol withdrawal symptoms. Abrupt cessation or reduction in alcohol use can trigger a stress response in the brain, leading to a sudden increase in anxiety and depression in alcohol-dependent individuals. Furthermore, long-term neurocognitive changes during withdrawal, resulting from the reward and stress systems, can increase the risk of depression among alcohol-dependent individuals (3).

Despite the link between alcohol withdrawal and depression, not all individuals experience depressive states when experiencing alcohol withdrawal due to differences in developmental plasticity among individuals. Genetic studies have linked these individual differences to specific allele variations caused by single nucleotide polymorphisms (SNPs) (4). However, these SNPs do not act in isolation and interact with environmental factors related to cognition, emotion, and behavior (5). Therefore, molecular genetics has become a frontier research topic exploring the mechanisms of genetic and environmental effects on individual differences in depression.

Major vault protein (MVP) is the major component (70%) of mammalian cellular ribonucleoprotein particles known as vaults. It forms vesicle structures throughout the cytoplasm and regulates activities related to vesicle transport. Abbondanza et al. reported that reduction of MVP may lead to malnutrition (6). Although there has been no reported association between MVP gene polymorphisms and depression in alcohol-dependent individuals, MVP plays an essential role in regulating nucleocytoplasmic transport, signaling transduction, cellular differentiation, cell survival, and many neurological diseases (7–12). Interestingly, the MVP is largely associated with psychiatric disorders such as temporal lobe epilepsy (TLE) and the individuals with TLE have a four to five times increased occurrence of depression (13). Besides, MVP has not been detected in glial cells or neurons of normal brain tissue; it is expressed in vascular endothelial cells, glial cells, and neurons at the lesion site of patients with drug-resistant temporal lobe epilepsy caused by hippocampal sclerosis, focal cortical dysplasia. Therefore, it is hypothesized that MVP is related to temporal lobe injury, and individuals with medial temporal lobe sclerosis or complex partial seizures are more likely to experience depressive symptoms (14, 15).

There have been few studies examining the details of the interaction between the environment and MVP gene polymorphisms. Two models can potentially explain the role of genetic factors in G × E interactions (gene–environment interaction). In the diathesis-stress model, carriers of the risk genotype variants, when exposed to adverse environmental experiences, would be more likely to develop the adverse outcome (16); the differential susceptibility model suggests that risk genotypes are considered plasticity or susceptibility genotypes and those carriers are susceptible to both adverse and enriched environments, for better and for worse (17).

In the present study, the rs4788186 polymorphism of MVP was used as a genetic indicator, and alcohol withdrawal was used as an environmental indicator to examine the moderating role of MVP rs4788186 polymorphism on the association between alcohol dependence and depression among Chinese male problem drinkers. Meanwhile, we explored the nature of G × E by comparing the diathesis stress with the differential susceptibility model.

## 2. Methods

### 2.1. Participants and procedure

Four hundred and thirty five male Chinese problem drinkers from several northern Chinese hospitals were recruited in this study. All the participants were patients hospitalized for alcohol use disorder, diagnosed by at least two trained psychiatrists based on the DSM-IV. Participants with a history of other substance abuse or dependence (excluding nicotine), cardiovascular, liver, or kidney disease, neurological disorders (e.g., epilepsy, multiple sclerosis), current infectious diseases (e.g., HIV, hepatitis B/C), and those with a personal or family history of severe psychiatric disorders (e.g., schizophrenia, bipolar disorder) were excluded. Participants who were unable to understand the informed consent form were also excluded. Demographic data, including age and years of education, were collected for all eligible participants.

Genomic DNA was extracted from 5 mL peripheral blood using the salting-out method from all the subjects (18). Buffy coats of nucleated cells obtained from anticoagulated blood (ACD or EDTA) were resuspended in 15 mL polypropylene centrifugation tubes with 3 mL of nuclei lysis buffer (10 mM Tris–HCl, 400 mM NaCl, and 2 mM Na_2_EDTA, pH 8.2). The cell lysates were digested overnight at 37°C with 0.2 mL of 10% SDS and 0.5 mL of a protease K solution (1 mg protease K in 1% SDS and 2 mM Na_2_EDTA). After digestion was complete, 1 mL of saturated NaCl (approximately 6 M) was added to each tube and shaken vigorously for 15 s, followed by centrifugation at 2,500 rpm for 15 min. The precipitated protein pellet was left at the bottom of the tube and the supernatant containing the DNA was transferred to another 15 mL polypropylene tube. Exactly 2 volumes of room temperature absolute ethanol were added and the tubes inverted several times until the DNA precipitated. The precipitated DNA strands were removed with a plastic spatula or pipette and transferred to a 1.5 mL microcentrifuge tube containing 100–200 μL TE buffer (10 mM Tris–HCl, 0.2 mM Na_2_EDTA, pH 7.5). The DNA was allowed to dissolve 2 h at 37°C before quantitation (19). MVP rs4788186 SNP was genotyped using the primers and probes for SNPs were analyzed using an TaqMan assay on-demand kit (C_2851919_20, ThermoFisher Scientific, Waltham, MA, United States). Reactions were performed according to the manufacturer’s protocols.

The participants were asked to complete a series of questionnaires and provide a blood sample for DNA extraction. The Institutional Review Board of the Inner Mongolian Medical University approved the study (Ethic approval number: YKD2015003). All staff involved in this study were trained before the study commenced. All patients were provided written informed consent, were asked to complete a series of questionnaires, and were told that the blood sample would be subjected to a gene assay.

### 2.2. Measures

#### 2.2.1. Assessment of alcohol dependence

Alcohol dependence level was measured using the Michigan Alcoholism Screening Test (MAST), a questionnaire containing a 25-item self-report in which respondents rate the severity of dependence-related alcohol use behaviors (20). Each of the 25 items on the MAST is rated on a four-point scale ranging from “not at all” (value = 0) to “extremely” (value = 4). The sum of the response scores ranges from 0 to 96. The scale has high internal consistency with a Cronbach’s *α* of 0.90.

#### 2.2.2. Assessment of depression

Depression was measured using the self-rating depression scale (SDS), which contains 20 items (21). Each item is rated on a four-point Likert scale (from 1 = “rarely or none of the time” to 4 = “most or all of the time”). Higher total scores indicate more severe symptoms of depression. The SDS has internal consistency with a Cronbach’s *α* of 0.79.

### 2.3. Statistical analysis

First, the *χ*^2^ test was used to determine whether the genotype distribution of MVP rs4788186 agreed with expectations and met Hardy–Weinberg equilibrium. Given the small number of subjects carrying the AG and GG genotypes, they were combined in subsequent analyses (AA = 245, AG and GG = 190). Pearson correlation study was used to determine the correlation between MVP rs4788186, age, years of education, level of alcohol dependence, and depression.

Second, we used traditional linear regression to examine the interaction effect of the MVP rs4788186 polymorphism with alcohol dependence on depression in male problem drinkers. When significant interactions were found, region of significance (RoS) analysis was applied to examine the nature of interaction effects. RoS analysis provides interval ranges where the association between MVP rs4788186 and the level of alcohol dependence is significant for estimating the forms of the G × E interaction.

Finally, a re-parameterized regression model was used to test the pattern of G × E interaction as follows (22):


$$Y=\{\begin{array}{c} \mathrm{Group}:D=0\;B_{0}+B_{1}\left(X-C\right)+B_{3}X_{2}+B_{4}X_{3}+E\\ \mathrm{Group}:D=1\;B_{0}+B_{2}\left(X-C\right)+B_{3}X_{2}+B_{4}X_{3}+E \end{array}$$

where *Y* is the outcome variable of depression, group is the different allelic group of MVP polymorphism, *X* is the MAST score, *X*_2_ and *X*_3_ are covariates (age and academic years), and C is the crossover point where the slopes of different genotypic subgroups cross, which is a critical parameter for judging the mode of interaction. If the point estimate and its 95% confidence interval fall at the maximum MAST score, the interaction fits the diathesis-stress model. Otherwise, the forms of interaction fit the differential susceptibility model. To elucidate the patterns of interaction, the models can be further subdivided into a strong/weak differential susceptibility model and a strong/weak diathesis-stress model. The strong model assumes that only individuals carrying the risk/plasticity allele are susceptible to environmental influences, while others carrying the non-risk/non-plasticity allele are unaffected by the environment. The weak model assumes that all individuals are affected by the environment, but some individuals with the risk/plasticity allele are more sensitive than those carrying the non-risk/non-plasticity allele. Thus, for nested models, the *F*-test is used to determine the most appropriate model, and for non-nested models, the Akaike information criterion (AIC) and Bayesian information criterion (BIC) are used to determine which model fits best.

## 3. Results

### 3.1. Descriptive statistics

The mean age of the participants was 43.60 years, with a standard deviation of 9.03, and the minimum and maximum ages were 20 and 67 years, respectively. The mean years of education were 10.95, with a standard deviation of 2.86, and the minimum and maximum years of education were 5 and 18, respectively. Descriptive statistics of research variables are shown in Table 1. The following is the genotype distribution of MVP rs4788186 in 435 male patients with AUD: 245 (56.32%) AA homozygotes, 168 (38.62%) AG homozygotes, and 22 (5.06%) GG homozygotes, which was consistent with Hardy–Weinberg equilibrium (*χ*^2^ = 0.99, *p* > 0.05). Given the small number of subjects carrying the AG and GG genotypes, they were combined and recoded in subsequent analyses (AG/GG = G allele = 0, AA homozygote = 1).

**Table 1:** Hardy–Weinberg equilibrium.

Genotype	Number of people	Percentage	
AA	245	56.32%	
AG	168	38.62%	
GG	22	5.06%	
*χ* ^2^	0.99	*p*	0.32

The independent sample t-test showed no significant difference between genotypic groups in MAST and depression scores (MAST: *t* = 0.33, *p* = 0.74; depression: *t* = −0.92, *p* = 0.36); see Table 2 for more details.

**Table 2:** Independent sample test.

rs4788186 polymorphism	Age	Educational years	Alcohol dependence	Depression
AA homozygote	44.16 ± 9.03	10.91 ± 2.75	9.14 ± 5.44	55.26 ± 11.68
G allele	42.89 ± 9.01	11.01 ± 3.00	8.96 ± 5.47	56.25 ± 10.44
*t*	1.45	−0.34	0.33	−0.92
*p*	0.15	0.73	0.74	0.36

The correlations among variables are shown in Table 3. No significant correlation between MVP rs4788186 and MAST or depression scores was observed. Age and depression scores were positively correlated with MAST scores (*r* = 0.23, *p* < 0.001) (*r* = 0.25, *p* < 0.001), while the years of education were negatively correlated with MAST (*r* = −0.23, *p* < 0.001).

**Table 3:** Descriptive statistics and correlations among study variables.

	rs4788186	Age	Educational years	Alcohol dependence	Depression
rs4788186	1				
Age	−0.08	1			
Educational years	0.02	−0.39^***^	1		
Alcohol dependence	−0.02	0.23^***^	−0.23^***^	1	
Depression	0.01	−0.2	−0.02	0.25^***^	1
*M*	(—)	43.60	10.95	9.06	55.69
SD	(—)	9.03	2.86	5.44	11.15

^***^p < 0.001.

### 3.2. The interactions of alcohol dependence level and MVP rs4788186 for depression

Traditional hierarchical regression analysis was conducted to identify the interaction between MVP rs4788186 and alcohol dependence level for depression. Alcohol dependence level significantly affected depression scores (*β* = 0.27, p < 0.001), such that a higher alcohol dependence level was associated with a higher depression level. There was no significant effect of MVP rs4788186 on depression (*β* = 0.04, *p* = 0.54). In the next step, the interaction of alcohol dependence level and MVP rs4788186 was included in the regression equation. The interaction of alcohol dependence level and MVP rs4788186 accounted for a significant portion of the variance in depression (*β* = −0.17, *p* < 0.05; Table 4).

**Table 4:** Interaction between rs4788186 and alcohol dependence on depression.

Variables	Depression					
	Δ*R^2^*	*B* (SE)	*β*	*t*	*p*	95%CI
Age	0.002	0.004 (0.01)	0.04	0.68	0.50	−0.01 to 0.02
Educational years		0.01 (0.02)	0.04	0.68	0.50	−0.02 to 0.05
Alcohol dependence	0.07	0.27 (0.05)	0.27	5.55	<0.001	0.17 to 0.36
rs4788186		−0.09 (0.09)	−0.04	−0.92	0.36	−0.27 to 0.10
Alcohol dependence × rs4788186	0.02	−0.25 (0.09)	−0.17	−2.71	0.01	−0.44 to −0.07

**Figure 1:**
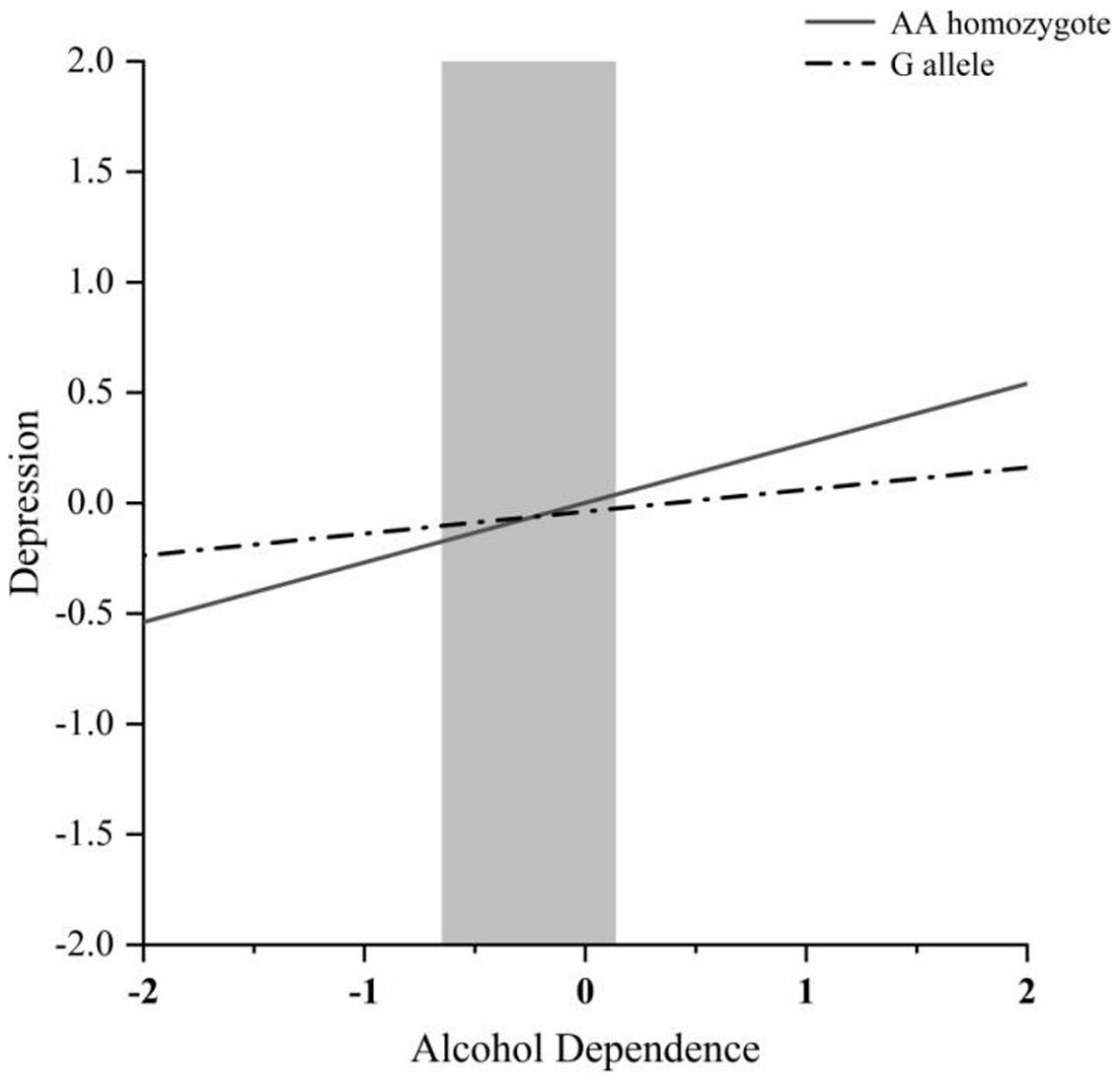
RoS test of the simple slopes on depression from alcohol dependence level in the MVP rs4788186 allelic group. The gray shaded area represents the 95% confidence interval of the crossover point C of the interaction on the alcohol use disorder severity axis, 95% confidence interval of the crossover point C ranging from −0.651 to 0.139. Simple slope at G allele = 0.10, *t* = 2.13, *p* = 0.034. Simple slope at AA homozygote = 0.27, *t* = 8.54, *p* = 0.000.

The RoS test can be performed to interpret the interaction effect (Figure 1). The simple slopes for alcohol dependence level on depression were as follows: AA homozygote carriers: *β* = 0.27, *t* = 8.54, *p* < 0.001; G allele carriers, *β* = 0.10, *t* = 2.13, *p* < 0.05; crossover point on predictor = −0.235. The lower and upper bounds of regions of significance were −0.651 and 0.139, respectively, suggesting that subjects with AA homozygotes would be more likely to experience high depression symptoms than subjects with the G allele.

### 3.3. Re-parameterized regression analysis

We performed a re-parameterized regression analysis to test a specific pattern of alcohol dependence level × MVP rs4788186 (Table 5). The weak differential susceptibility (Model B) explained a significant amount of variance in depression (*R*^2^ = 0.08, *p* < 0.001; Table 5). The crossover point C estimated = −0.34 (SE = 0.39), where the slopes from alcohol dependence level to depression in the G allele group (*B*_1_ = 0.13, SE = 0.07) and AA homozygote group (*B*_2_ = 0.38, SE = 0.06, *p* < 0.001) were significant. Based on Model B, constraining *B*_1_ = 0 led to Model A (strong differential susceptibility); fixed C to the maximum of MAST scores led to model C (strong diathesis-stress) and Model D (weak diathesis-stress). This study used the *F*-test with AIC and BIC values for model comparison. Compared with Model B, Model A reduced one parameter and explained a similar variance (Δ*R*^2^ = 0.00, *F* = 3.12). Compared with Model A, Model C reduced one parameter and explained less variance (Δ*R*^2^ = 0.05, *F* = 23.43, *p* < 0.001). Because Model A and Model D are non-nested models, we compared the values of AIC and BIC. The AIC and BIC values of Model A were relatively smaller than that of Model D, lending support for Model A (the strong differential susceptibility model). All statistical indexes supported Model A (i.e., strong differential susceptibility), in which the G allele was a non-plasticity allele, and the AA homozygote was a plasticity homozygote.

**Table 5:** Results for re-parameterized regression model for depression.

Parameter	Differential susceptibility		Diathesis-stress	
	Strong: Model A	Weak: Model B	Strong: Model C	Weak: Model D
*B*_0_	−0.40 (0.37)	−0.43 (0.38)	−0.25 (0.38)	0.04 (0.37)
*B*_1_	(—)	0.13 (0.07)	(—)	0.25^***^ (0.06)
*C*	−0.23 (0.25)	−0.34 (0.39)	1.48 (—)	1.48 (—)
95%CI of *C*	(−0.72, 0.26)	(−1.10, 0.42)	(—)	(—)
*B*_2_	0.37^***^ (0.06)	0.38^***^ (0.06)	0.16^***^ (0.05)	0.29^***^ (0.05)
*B*_3_	0.01 (0.01)	0.01 (0.01)	0.01 (0.01)	0.01 (0.01)
*B*_4_	0.002 (0.02)	−0.001 (0.02)	0.01 (0.02)	−0.02 (0.02)
*R* ^2^	0.08	0.08	0.03	0.07
*F* (d*f*)	9.21^***^ (4,430)	9.24^***^ (5,429)	4.25^***^ (3,431)	8.00^***^ (4,430)
*F* vs. *A*(d*f*)	(—)	3.12 (1,428)	23.43^***^ (1,429)	(—)
*F* vs. (d*f*)	3.12 (1,428)	(—)	13.33^***^ (2,427)	7.66^***^ (1,428)
AIC	1209.72	1208.57	1230.80	1214.26
BIC	1234.17	1237.09	1251.17	1238.72

^***^p < 0.001.

## 4. Discussion

We examined the single and interacting effects of alcohol dependence and rs4788186 polymorphism on depression. Hardy–Weinberg testing showed that rs4788186 was stably inherited in the population, and independent sample *t*-tests showed that the rs4788186 polymorphism did not significantly affect alcohol-dependent severity or depression scores alone. In contrast, the severity of alcohol dependence was significantly and positively associated with depression. Two-factor interaction analysis showed that alcohol dependence severity and rs4788186 polymorphism acted simultaneously on depression. In the G × E model, the interaction between alcohol dependence and rs4788186 polymorphism was consistent with various susceptibility models. Specifically, rs4788186 AA carriers had higher susceptibility to depression than G carriers in a non-drinking environment and had higher depression scores in a drinking environment and lower depression scores in a non-drinking environment.

The close relationship between alcohol dependence and depression has been confirmed (23). We also found a significant positive relationship between alcohol dependence scores and depression (*β* = 0.25, *p* < 0.001). Researchers believe that heavy chronic or binge alcohol exposure causes severe debilitating diseases in the central nervous system, comprising the frontal and temporal cortex, hippocampal, cerebellum, insula, and brainstem (24, 25). This phenomenon leads to decreased brain function (i.e., planning, verbal fluency, memory, cognition, and emotion) (26). A meta-analysis reported smaller temporal lobe and hippocampal volumes in depression patients (12, 27, 28), suggesting that the positive relationship between alcohol exposure and depression is connected with impairment of the temporal lobe. However, there was no difference between rs4788186 AA/G carriers regarding alcohol dependence and depression scores (*t* = 0.33, *p* = 0.74; *t* = −0.92, *p* = 0.36, Table 2).

In mammals, MVP is the primary component (70%) of cellular ribonucleoprotein particles known as vaults and is implicated in regulating several cellular processes, including nucleocytoplasmic transport, signaling transduction, cellular differentiation, cell survival, and immune responses. Previous studies of MVP focused on tumors, drug resistance, and epilepsy (29–31); however, the roles of MVP in alcohol dependence or depression have not been reported. Two studies of the rs4788186 polymorphism on drug resistance reported negative results (32, 33), suggesting that the unitary effect of rs4788186 polymorphism might be insufficient to influence the severity of depression. However, with alcohol exposure, we found a significant effect of MVP rs4788186 on alcohol dependence and depression (*β* = −0.17, *p* < 0.05; Table 4).

MVP is closely related to temporal lobe epilepsy (29). Major depressive disorder is the most common comorbid psychiatric condition associated with temporal lobe epilepsy (34). Therefore, we suggest that the interaction between alcohol and MVP on depression is most likely to change the functions of the temporal lobe. In this study, rs4788186 G carriers showed lower depression scores in alcohol exposure and higher depression scores in non-alcohol exposure than AA carriers. The result is consistent with the different susceptibility models of the gene–environment interaction.

An imaging study showed significant temporal lobe volume deficits in cortical gray matter, white matter, and anterior hippocampus in chronic alcoholic men relative to controls (35). MVP plays an essential role in vesicular transport (36), while rs4788186 G mutation leads to an impairment of transport function in alcohol exposure, and the temporal lobe dysfunction was alleviated. In non-alcohol exposure, the rs4788186 G mutation affected nutrient transportation and led to the atrophy of temporal lobes6, as previous researchers reported smaller temporal lobes and hippocampal volumes in depression patients (12, 27, 28).

This study was the first to identify an interaction between rs4788186 and alcohol exposure on depression, consistent with various susceptibility models. However, we acknowledge that our research has several limitations that need to be considered. Firstly, we did not take into account several crucial factors, such as social support, family history and comorbidities, which may have influences on the development of depression in individuals with alcohol dependence. Secondly, our study relied on self-report measures to assess depression, which may have introduced subjective bias and affected the accuracy of the results. Additionally, our sample includes individuals who have depressive symptoms while have no confirmed diagnosis of depression, which limits the generalizability of the findings to the broader population of individuals with depression. Finally, our study was cross-sectional in nature, which precludes any causal inferences between the variables examined. These limitations highlight the need for further research to address these factors and exercise caution when interpreting the findings of this study.

## 5. Conclusion

This study, which analyzed a specific gene–environment interaction, demonstrated that carriers of the AA homozygote of MVP rs4788186 may be more susceptible to severe alcohol problems and higher levels of depression during withdrawal.

## Data availability statement

The original contributions presented in the study are included in the article/supplementary material, further inquiries can be directed to the corresponding authors.

## Ethics statement

The studies involving human participants were reviewed and approved by The Institutional Review Board of the Inner Mongolian Medical University approved the study. The patients/participants provided their written informed consent to participate in this study.

## Author contributions

YW, YC, FQ,YY, and LW: data collection, literature review, and wrote the first draft of the manuscript. KZ, YK, and YJ: wrote sections of the manuscript. GS and KW: performed statistical analysis. WW, LC, YL, FW, XP, and LX contributed to conception and design of the study. All authors read and approved the final manuscript.

## Funding

This study was supported by the Natural Science Foundation of Xinjiang Province (2018D01C239), Project of NINGBO Leading Medical & Health Discipline (2022-X27, LX), and Project of Medical Technology of Zhejiang Province (2016A610016, 2020KY908, LX).

## Conflict of interest

The authors declare that the research was conducted in the absence of any commercial or financial relationships that could be construed as a potential conflict of interest.

## Publisher’s note

All claims expressed in this article are solely those of the authors and do not necessarily represent those of their affiliated organizations, or those of the publisher, the editors and the reviewers. Any product that may be evaluated in this article, or claim that may be made by its manufacturer, is not guaranteed or endorsed by the publisher.
